# Skeletal Muscle Insulin Resistance in Three Dimensions: Mechanisms, Myokines, and Microphysiological Platform

**DOI:** 10.3390/cells15201892

**Published:** 2026-10-17

**Authors:** Mohamed Salih, Joe Bacon, Simon C. Johnson, Gerrit Hilgen, James Henstock, Davina C. M. Simoes

**Affiliations:** Faculty of Science and Environment, Northumbria University, Newcastle upon Tyne NE1 8ST, UK; salih.mohamed@northumbria.ac.uk (M.S.); joe.bacon@northumbria.ac.uk (J.B.); simon.c.johnson@northumbria.ac.uk (S.C.J.); gerrit.hilgen@northumbria.ac.uk (G.H.); james.henstock@northumbria.ac.uk (J.H.)

**Keywords:** three-dimensional engineered muscles, myokines, GLUT4, extracellular matrix mechanobiology, type 2 diabetes, electrical pulse stimulation, lipotoxicity, irisin, phosphoproteomics, precision metabolic medicine

## Abstract

**Highlights:**

**What are the main findings?**

- Three-dimensional (3D) engineered muscle tissues (EMTs) can reproduce key features of skeletal muscle insulin resistance within a single integrated construct, including lipid-induced insulin resistance, myosteatosis, measurable contractile force and contraction-regulated myokine secretion, thereby complementing conventional 2D in vitro models.
- 3D co-culture systems can capture aspects of tissue crosstalk and endocrine function, demonstrating that inflammatory adipose-muscle interactions can drive metabolic reprogramming and contractile impairment.

**What are the implications of the main findings?**

- Current models remain limited by incomplete fibre maturation and the absence of physiological processes such as innervation and fluid perfusion. Adding these features could improve their physiological fidelity.
- Coupling more complex 3D models with functional phenotyping, multi-omics, and myokine profiling may support mechanistic drug screening and patient-specific disease modelling, offering a potential route toward precision metabolic medicine.

**Abstract:**

Skeletal muscle insulin resistance is a major determinant of impaired glucose disposal in type 2 diabetes. The mechanisms underlying insulin resistance in muscles are shaped by tissue architecture, contraction, extracellular-matrix signalling, mitochondrial function, and endocrine communication; processes that are not fully reproduced by conventional cell-based two-dimensional (monolayer) models of disease. This narrative review integrates our current understanding of canonical IRS-1–PI3K–AKT–GLUT4 signalling with contraction-dependent pathways, lipotoxicity, mitochondrial and endoplasmic-reticulum stress, inflammation, extracellular-matrix remodelling, and myokine dysregulation. We then evaluate experimental models, with particular emphasis on three-dimensional (3D) engineered muscle tissues (EMTs). Current evidence indicates that 3D EMTs can provide aligned myofibres, matrix-dependent mechanosignalling, measurable force and calcium handling, insulin-stimulated glucose uptake, and contraction-regulated myokine secretion. Emerging co-culture and myosteatosis models also recapitulate aspects of adipose–muscle crosstalk. However, current platforms remain limited by incomplete fibre maturation, absent or immature neuromuscular junctions, limited vascularisation and perfusion, variability across cell and scaffold systems, and sparse validation against patient-specific multi-omic signatures. Integrating patient-derived cells, innervation, perfusion, controlled mechanics, and multi-tissue modules could make 3D EMTs a more predictive microphysiological platform for mechanistic studies, therapeutic screening, and precision metabolic medicine.

## 1. Introduction

Skeletal muscle presents a fundamental paradox in the study of metabolic disease. It is the body’s largest insulin-responsive tissue and a principal determinant of postprandial glucose clearance, yet it remains comparatively under-represented in human microphysiological (in vitro) disease modelling. This reflects two converging factors: the intrinsic complexity of skeletal muscle as a terminally differentiated, mechanically loaded tissue that depends on innervation and vascularisation to maintain its mature metabolic phenotype; and a longstanding research bias prioritising pancreatic β-cell dysfunction as the primary lesion in type 2 diabetes mellitus (T2DM). Together, these factors have left the importance of skeletal muscle comparatively understudied as an early contributor to insulin resistance [1,2].

Muscle insulin sensitivity is shaped by a set of interdependent features that, in vivo, collectively determine how a myofibre responds to insulin. These include fibre-type composition [3], mitochondrial oxidative capacity [4], extracellular matrix (ECM) organisation [5], and contractile mechanical demand [6]. Because these features interact, rather than operate in isolation, disruption of any one of them can impair glucose disposal and precipitate insulin resistance [2]. Understanding how their disruption drives systemic metabolic disease therefore requires experimental systems capable of preserving this biological complexity.

Skeletal muscle is now recognised as an endocrine organ that secretes a dynamic repertoire of bioactive peptides and cytokines known as myokines in response to contraction, metabolic stress, and inflammatory challenge [7]. In healthy skeletal muscle, myokines mediate the beneficial systemic effects of exercise by promoting insulin sensitivity, mitochondrial biogenesis, fatty acid oxidation, and anti-inflammatory signalling in muscle, liver, adipose tissue, the brain and retina [8,9]. In insulin resistance, the myokine secretome undergoes a characteristic dysregulation [10]. Beneficial contraction-linked myokines, including irisin and apelin, are suppressed, while pro-atrophic and pro-inflammatory myokines, including myostatin, are elevated. These changes transform skeletal muscle from a metabolic ally in glucose homeostasis into an active propagator of systemic dysfunction [10,11]. The myokine dimension of insulin resistance has been largely inaccessible to research using conventional in vitro models, which lack the contractile architecture necessary for physiological myokine secretion [12].

Human skeletal muscle biopsies, animal models, and conventional 2D myotube cultures have each contributed substantially to defining these mechanisms, but no single system captures the full combination of the structural organisation, contractile function, and dynamic metabolic environment of human skeletal muscle in vivo [2,13]. Three-dimensional engineered skeletal muscles now offer a complementary approach to the traditional study systems and allow integration of several of these factors into a single model, enabling combinatorial effects to be studied in vitro.

The terminology in this field is used variably, and it is useful to distinguish three levels of complexity: Engineered muscle tissues (EMTs) comprise myogenic cells that self-organise around biomechanical anchors within extracellular-matrix scaffolds to form aligned, striated, contractile myofibres [14]; organoids, which are self-organising multicellular structures derived from stem cells; and microphysiological systems (MPS) or organ-on-chip platforms, which add features such as multiple tissue types, microfluidic perfusion, or vascularisation to reproduce tissue- and organ-level function with greater fidelity. These differ substantially in biological and technical complexity, and the translational claims that can reasonably be made of a model scale with that complexity. This distinction is maintained throughout this review, in which “EMT” denotes engineered muscles, and “MPS” the more complex culture systems.

Unlike 2D monolayer cultures, EMTs can integrate contractile function, matrix mechanosignalling, and a measurable, contraction-regulated myokine secretome within a single construct [12,15], a capacity that motivates their application to insulin resistance.

The canonical framework of skeletal muscle insulin resistance has centred on impaired phosphorylation of insulin receptor substrate-1 (IRS-1), reduced phosphoinositide 3-kinase (PI3K)–protein kinase B (AKT) signalling, and defective translocation of glucose transporter type 4 (GLUT4) to the plasma membrane, which together limit insulin-stimulated glucose uptake and glycogen synthesis [16]. This framework is now being substantially revised. Personalised phosphoproteomic profiling has demonstrated that insulin resistance involves selective disruptions to non-canonical signalling networks extending well beyond canonical AKT targets, revealing molecular heterogeneity that population-level disease classifications do not capture [16,17]. This molecular heterogeneity, extending from canonical IRS-1/AKT signalling through non-canonical regulators such as the deubiquitinase MINDY1 [16,17] and through the dysregulated myokine secretome [10,18], demands experimental systems capable of capturing individualised muscle phenotypes within functionally organised tissue. To our knowledge, no prior review has examined what 3D EMTs reveal about both the intracellular and myokine-mediated dimensions of insulin resistance, or how these platforms might bridge the translational gap between molecular phenotyping in human tissue and functional in vitro disease modelling.

This narrative review addresses that gap. We begin by describing the mechanisms through which skeletal muscle becomes insulin resistant, integrating recent phosphoproteomic and multi-omic insights with established metabolic, structural, and endocrine biology. We then briefly survey conventional experimental models and their limitations before examining 3D EMTs and the functional assays that make them suited to insulin resistance research. The core of the review evaluates, mechanism by mechanism, what 3D EMTs have uniquely revealed, including their capacity to capture the contraction-regulated myokine secretome and mechanistically relate this to dysregulation in insulin resistance. We conclude by identifying the technical gaps and translational barriers that must be resolved for these systems to fulfil their full potential as predictive models.

## 2. Mechanisms of Glucose Uptake in Healthy Skeletal Muscle

### 2.1. Skeletal Muscle in Glucose Homeostasis

Skeletal muscle is the dominant site of peripheral glucose disposal, accounting for approximately 80–90% of insulin-stimulated glucose uptake measured under hyperinsulinaemic-euglycaemic clamp conditions and is therefore the principal effector tissue determining whether circulating glucose is cleared or remains elevated [19,20]. Because muscle handles the majority of an insulin-stimulated glucose load, even modest defects in muscle insulin responsiveness contribute substantially to systemic glucose dysregulation, whether affecting signalling efficiency, glucose transporter trafficking, or mitochondrial substrate handling. Although T2DM is a multi-organ disorder involving the liver, adipose tissue, pancreatic islets, gut, and brain, skeletal muscle insulin resistance is an early and important contributor to its development [20,21].

Muscle metabolic function is shaped by its fibre-type composition. Human skeletal muscle contains slow oxidative type I fibres, fast oxidative–glycolytic type IIa fibres, and fast glycolytic type IIx fibres, in proportions that vary between muscle groups and individuals according to genetics, sex, age, and habitual physical activity [22,23]. Oxidative type I and type IIa fibres generally exhibit higher mitochondrial density, oxidative capacity, capillarisation, GLUT4 expression, and insulin sensitivity than glycolytic type IIx fibres, which show greater susceptibility to lipid-induced metabolic dysfunction [24,25]. These associations are relative rather than absolute and the relationship between fibre type and insulin sensitivity should not be interpreted as strictly unidirectional [26]. In obesity and physical inactivity, a shift towards a more glycolytic profile is often observed [26,27], whereas ageing and sarcopenia are associated with a different pattern. There is preferential loss of type IIx fibres, alongside declining oxidative capacity in the remaining fibres. Thus, metabolic risk accumulates through fibre-specific functional deterioration rather than a single, uniform shift [27,28,29]. The shared consequence across these contexts is a reduction in oxidative fibre quality and mitochondrial capacity, linking fibre-type biology to the mitochondrial and lipotoxic mechanisms discussed in Section 2.4.

### 2.2. Canonical and Non-Canonical Pathways of Glucose Uptake

**Figure 1:**
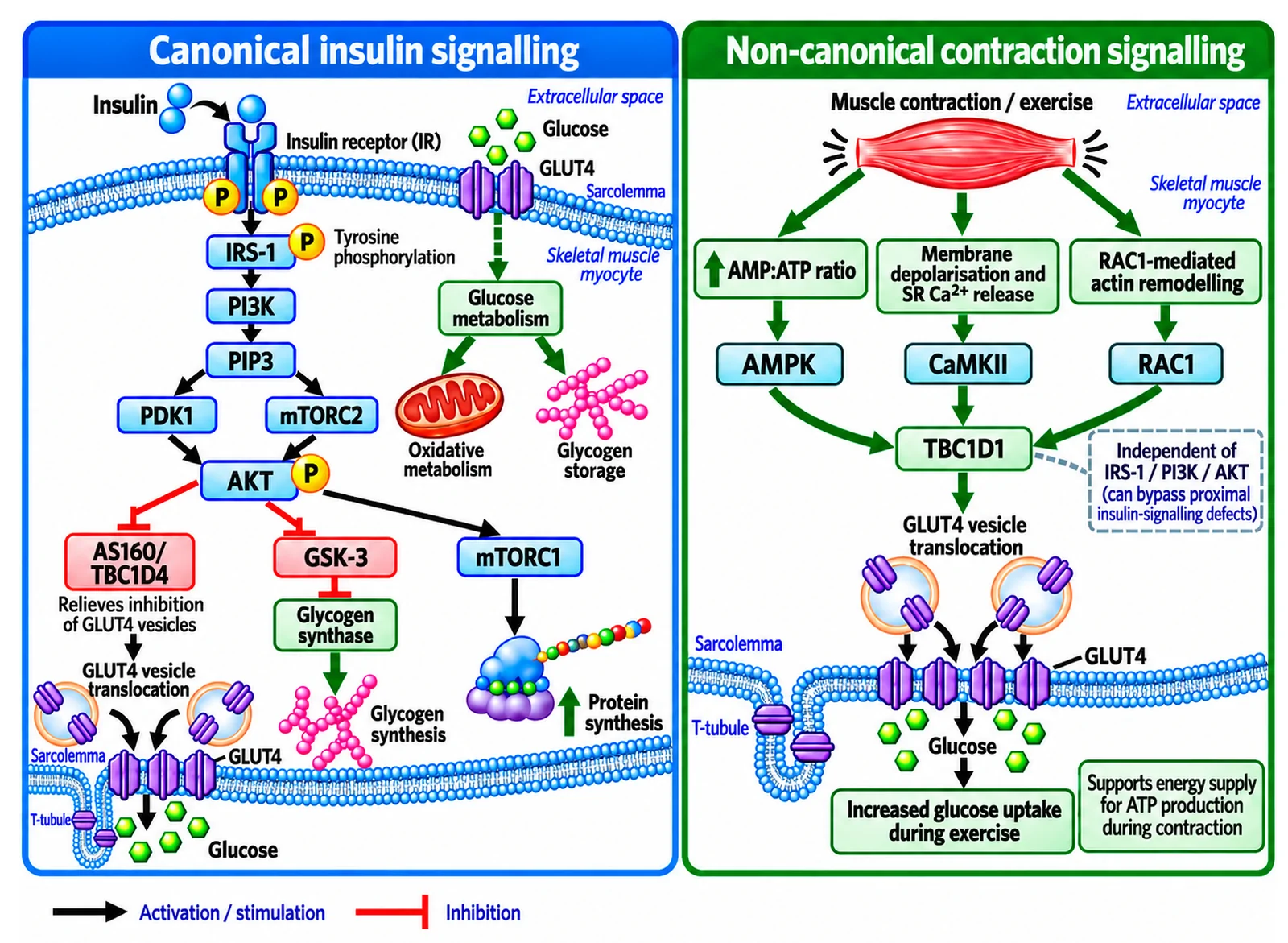
Canonical and non-canonical pathways of insulin-stimulated glucose uptake in skeletal muscle. Canonical pathway (**Left** panel): insulin binding to the insulin receptor (IR) triggers sequential tyrosine phosphorylation of IRS-1, activation of PI3K, PIP3 generation, and PDK1-mediated AKT phosphorylation. Activated AKT inactivates AS160 (TBC1D4), releasing GLUT4 storage vesicles from the perinuclear cytoplasm to translocate to the sarcolemma and T-tubules, increasing glucose transport capacity. Imported glucose is directed into glycogen synthesis or oxidative metabolism; AKT additionally activates mTORC1 to support protein synthesis. Non-canonical pathway (**Right** panel): during muscle contraction, two distinct inputs converge on TBC1D1 (an AS160 paralogue) to drive GLUT4 translocation independently of IRS-1, PI3K, or AKT. An increased AMP:ATP ratio activates AMPK, while depolarisation-induced calcium release from the sarcoplasmic reticulum activates calcium/calmodulin-dependent protein kinase (CaMKII). This insulin-independent pathway bypasses proximal signalling defects in insulin resistance and restores glucose uptake for up to 48 h following a single bout of exercise. Arrows: solid pointed arrow, activation/stimulation; red blunt-ended (⊣), inhibition.

In healthy muscle, glucose uptake is principally governed by insulin-stimulated translocation of GLUT4 to the sarcolemma and transverse (T) tubules (Figure 1). Insulin binding to the insulin receptor (IR) triggers receptor autophosphorylation and tyrosine phosphorylation of IRS-1, generating docking sites that recruit the p85 regulatory subunit of PI3K. PI3K generates phosphatidylinositol-3,4,5-trisphosphate (PIP3), recruiting 3-phosphoinositide-dependent protein kinase-1 (PDK1), which phosphorylates AKT at Thr308, while mammalian target of rapamycin complex 2 (mTORC2) independently phosphorylates AKT at Ser473. Both events are required for full AKT activation [30]. Activated AKT phosphorylates and inactivates AS160 (TBC1D4), a Rab-GTPase-activating protein that otherwise retains GLUT4 storage vesicles in the perinuclear cytoplasm. Inactivation of AS160 releases these vesicles for translocation and fusion with the sarcolemma and T tubules, markedly increasing membrane glucose transport capacity [30,31]. Imported glucose is directed into glycogen synthesis via AKT-mediated inhibition of glycogen synthase kinase-3 (GSK-3), which disinhibits glycogen synthase (GS) and promotes glycogen deposition, a process that accounts for the majority of non-oxidative glucose disposal in the postprandial state and is among the earliest metabolic defects detectable in insulin-resistant muscle [32]. The remainder enters glycolysis and mitochondrial oxidative phosphorylation to support ATP production. In parallel, AKT activates mTORC1 to promote protein synthesis and muscle hypertrophy, and suppresses FoxO-dependent transcription of atrogenes, linking insulin signalling to both metabolic and anabolic muscle function [30,33].

A second, insulin-independent mechanism of GLUT4 translocation is activated by muscle contraction and energy stress through distinct proximal signalling pathways (Figure 1). An increased AMP:ATP ratio activates AMP-activated protein kinase (AMPK), which phosphorylates TBC1D1, a paralogue of AS160, releasing a distinct pool of GLUT4 vesicles independently of IRS-1 or AKT [34]. Concurrently, depolarisation-induced ryanodine receptor-mediated calcium release from the sarcoplasmic reticulum activates calcium/calmodulin-dependent protein kinase II (CaMKII) [34,35]. A third contraction-activated signal, RAC1, promotes translocation through actin cytoskeleton remodelling [1]. This contraction-linked pathway contributes to the ability of a single exercise bout to enhance insulin-stimulated glucose uptake for up to 48 h, through AMPK-driven trafficking together with increased GLUT4 expression, improved mitochondrial capacity, and reduced intramyocellular lipid [16,35]. Because it bypasses proximal IRS-1 and AKT defects, the contraction-AMPK-TBC1D1 pathway represents a potential therapeutic target. However, its mechanistic importance should not be equated with therapeutic validation. The same contraction signals also trigger the release of insulin-sensitising myokines, linking the exercise-glucose uptake axis with the endocrine function of muscle [36].

Large-scale phosphoproteomic profiling has identified additional non-canonical regulators. Personalised profiling of human skeletal muscle across a spectrum of insulin sensitivity shows that insulin resistance involves selective and time-dependent disruptions extending well beyond canonical AKT targets, including impaired mTORC1 responses [16]. Integrating these data with genome-wide association data for glycaemic traits identified MINDY1, a lysine-48-specific deubiquitinase, as a direct AKT substrate. Phosphorylation at Ser441 activates its ubiquitin ligase activity, while MINDY1 knockdown enhances insulin-stimulated glucose uptake, establishing it as a negative regulator of insulin action [16,37]. MINDY1 exemplifies a class of regulatory nodes that may be invisible to canonical pathway analysis, placing insulin sensitivity at the intersection of signal transduction and proteostasis. Such nodes may differ between individuals with clinically similar degrees of glucose intolerance, providing a molecular basis for the heterogeneity of insulin resistance phenotypes across populations [16,38].

### 2.3. How Skeletal Muscle Becomes Insulin Resistant

**Figure 2:**
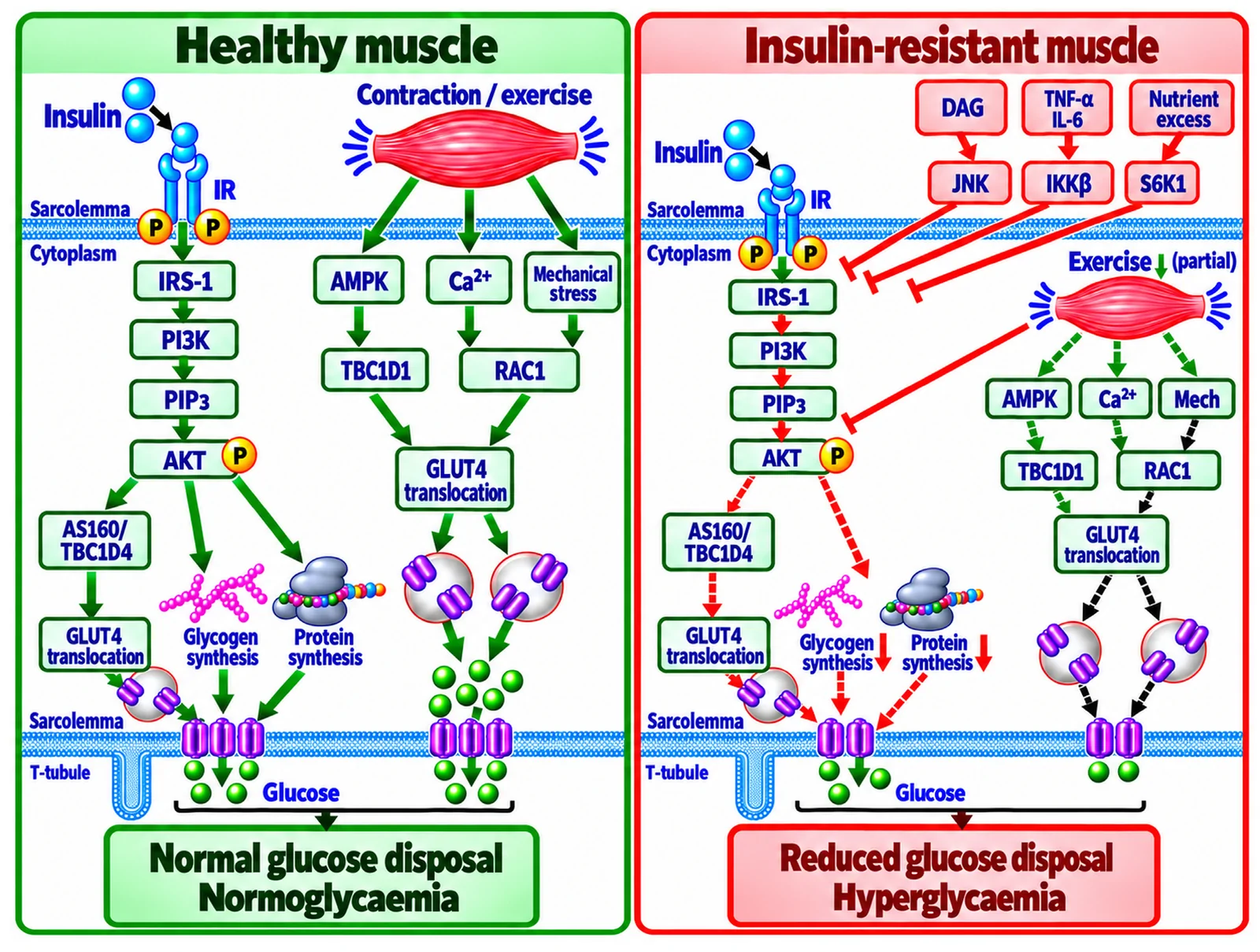
Healthy versus insulin-resistant skeletal muscle insulin signalling. (**Left** panel): in healthy skeletal muscle, insulin binding activates IR and IRS-1, leading to intact PI3K–AKT signalling and efficient GLUT4 translocation to the plasma membrane, resulting in normal glucose uptake and normoglycaemia. (**Right** panel): skeletal muscle with insulin resistance, converging stress kinases (JNK, IKKβ, S6K1) promote inhibitory serine phosphorylation of IRS-1, reducing PI3K–AKT activation, limiting GLUT4 translocation, and decreasing insulin-stimulated glucose uptake, contributing to hyperglycaemia. Arrows: green, activation/stimulation; red blunt-ended (⊣), inhibition; dashed line, reduced signalling/translocation; including GLUT 4 translocation, glycogen synthesis, and protein synthesis; black solid, downstream cellular processes.

In insulin-resistant skeletal muscle, both the canonical and non-canonical arms described above are impaired, though through partly distinct mechanisms and with different degrees of reversibility [16,28]. Within the canonical pathway, the activating tyrosine phosphorylation of IRS-1 that initiates PI3K–AKT signalling is antagonised by inhibitory serine/threonine phosphorylation at Ser307 and Ser636/639 (Figure 2), driven by converging stress kinases including c-Jun N-terminal kinase (JNK), inhibitor of κB kinase β (IKKβ), and mTORC1 acting through S6 kinase 1 (S6K1) [39]. These kinases are themselves activated by lipid intermediates, inflammatory cytokines, and nutrient excess (Section 2.4). This uncouples IRS-1 from PI3K, reducing PIP3 generation and AKT activation, with three downstream consequences: reduced AS160 phosphorylation retains GLUT4 vesicles in the perinuclear compartment, lowering sarcolemmal glucose uptake; impaired GSK-3 inhibition reduces glycogen synthase activity, disrupting this non-oxidative glucose disposal; and reduced mTORC1 activation limits anabolic signalling, contributing to the muscle atrophy that accompanies chronic insulin resistance [16,30,33].

Within the non-canonical pathway, insulin resistance does not abolish AMPK- or RAC1-mediated GLUT4 translocation (Figure 2), which is why acute exercise remains effective even in established T2D [16,35]. However, chronic insulin resistance progressively blunts this compensatory capacity, as mitochondrial oxidative capacity declines, AMPK activation is impaired, TBC1D1 phosphorylation is reduced, and RAC1-mediated actin remodelling is disrupted [1,6,16,35]. The result is a state of dual pathway insufficiency in which neither insulin- nor contraction-mediated GLUT4 translocation can fully restore glucose clearance, providing a mechanistic basis for the clinical observation that combined pharmacological and lifestyle interventions are more effective than either alone [6].

Crucially, the molecular architecture of insulin resistance is not uniform. Individual skeletal muscle proteome and phosphoproteome profiles predict whole-body insulin sensitivity more strongly than disease status alone, with marked interindividual variation in the specific signalling nodes that are disrupted [16,17]. This heterogeneity, extending from canonical IRS-1/AKT signalling through non-canonical regulators such as MINDY1, has direct implications for experimental modelling. Systems that resolve these individual molecular signatures within functional human muscle tissue, rather than averaging across population-level phenotypes, are needed to advance mechanistic understanding and therapeutic precision. This requirement motivates the evaluation of experimental models that follows [17].

### 2.4. Additional and Integrative Mechanisms of Insulin Resistance

**Figure 3:**
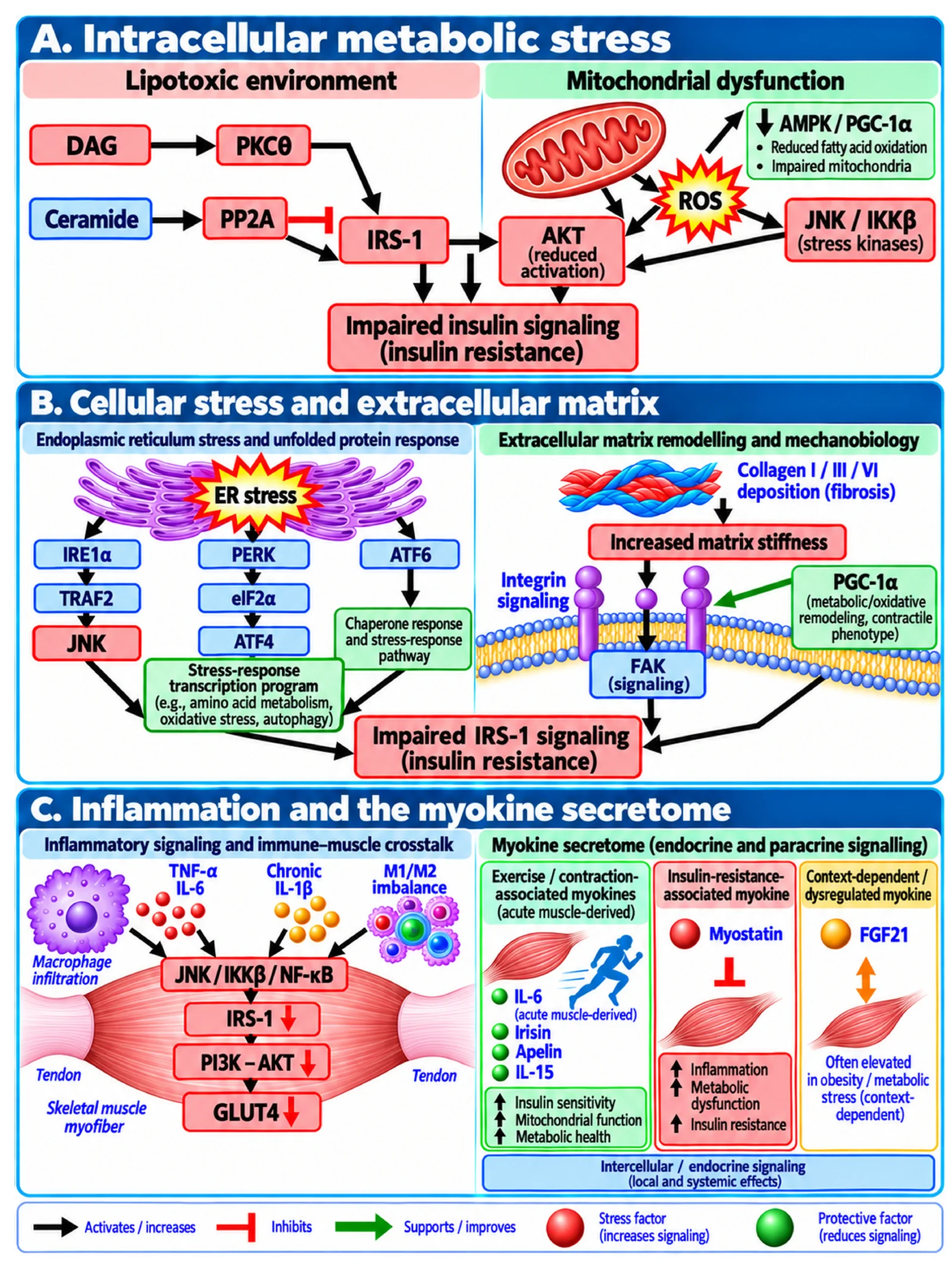
Integrated mechanisms of skeletal muscle insulin resistance. Mechanisms are grouped into three panels. (**A**) Intracellular metabolic stress. Lipotoxic environment (**Left** panel): under chronic nutrient excess, diacylglycerol (DAG) activates protein kinase C theta (PKCθ), driving inhibitory IRS-1 serine phosphorylation, while ceramides suppress AKT activation through protein phosphatase 2A (PP2A). Mitochondrial dysfunction (**Right** panel): lipid oversupply and incomplete β-oxidation generate reactive oxygen species (ROS), which activate the stress kinases JNK and IKKβ; while reduced AMPK/PGC-1α activity impairs fatty acid oxidation and mitochondrial function. Both arms converge on impaired insulin signalling. (**B**) Cellular stress and extracellular matrix. Endoplasmic reticulum (ER) stress and the unfolded protein response (**Left** panel): under conditions of nutrient excess, the IRE1α–TRAF2-JNK and PERK–eIF2α-ATF4 signalling pathways are activated, triggering a cellular stress-response transcriptional programme. ECM remodelling and mechanobiology (**Right** panel): increased deposition of collagens I, III, and VI raises matrix stiffness, enhancing integrin–focal adhesion kinase (FAK) signalling. Stiffness-associated suppression of PGC-1α further links matrix remodelling to impaired metabolic function, oxidative capacity and contractile phenotype. Together, these mechanisms contribute to impaired IRS-1 signalling. (**C**) Inflammation and the myokine secretome. Inflammatory signalling and immune–muscle crosstalk (**Left** panel): macrophage infiltration, M1/M2 polarisation imbalance and myocyte-intrinsic stress contribute to elevated TNF-α, IL-1β, and IL-6. These inflammatory mediators activate JNK/IKKβ/NF-κB signalling, promoting inhibitory IRS-1 serine phosphorylation, reduced PI3K–AKT signalling, and decreased GLUT4 translocation. Myokine secretome (**Right** panel): under healthy conditions, exercise-associated myokines (irisin, apelin, IL-6, IL-15) support insulin sensitivity, mitochondrial function, and metabolic health. In insulin resistance states, the myokine profile becomes dysregulated, with altered myostatin and FGF21 signalling potentially contributing to inflammation, adiposity, metabolic dysfunction, and insulin resistance. Arrows: black, activation/increase; red, stress/damage signalling; red blunt-ended (⊣), inhibition.

*Skeletal muscle* insulin resistance rarely develops in isolation (a notable exception being mutations in muscle-specific isoforms of TBC1D4, which cause severe primary muscle insulin resistance [1]), and it forms a part of a broader disease network that links T2DM, cardiovascular disease, metabolic dysfunction-associated steatotic liver disease (MASLD), and chronic kidney disease [40,41,42]. Several interacting mechanisms converge on the canonical and non-canonical signalling defects described above and understanding them defines the criteria that any experimental models must satisfy (Figure 3).

*Lipotoxicity*. Under chronic nutrient excess, intramyocellular triglycerides (IMTGs) accumulate and serve as a reservoir for bioactive lipid intermediates diacylglycerols (DAGs) and ceramide (Figure 3). Their lipotoxic potency depends more on the subcellular localisation and species composition, rather than total abundance [43]. Membrane-associated DAG activates protein kinase C theta (PKCθ), driving the inhibitory IRS-1 serine phosphorylation and PI3K uncoupling described in Section 2.3, while ceramides independently suppress AKT activation through protein phosphatase 2A [44,45,46]. This lipid overflow is not confined to muscle: similar adipose-derived flux underlies hepatic steatosis in MASLD, linking muscle lipotoxicity to the wider disease network [42].

*Mitochondrial dysfunction*. When fatty acid delivery exceeds oxidative capacity, incomplete β-oxidation generates acylcarnitines and reactive oxygen species (ROS) [47,48] (Figure 3). ROS and medium/long-chain acylcarnitines activate the same JNK and IKKβ stress kinases that drive IRS-1 serine phosphorylation, reinforcing the signalling defect [48,49]. Reduced AMPK activity compounds this defect by suppressing PGC-1α-dependent mitochondrial biogenesis and blunting the contraction-linked glucose uptake pathway, while impaired mitochondrial dynamics sustain a self-amplifying cycle of lipid accumulation and ROS generation [50]. Because PGC-1α also drives expression of FNDC5 (the precursor irisin), its suppression links mitochondrial dysfunction directly to the myokine changes discussed below.

*Inflammation.* Both myocyte-intrinsic stress (lipid, ROS, and ER stress) and paracrine signalling from infiltrating macrophages amplify insulin resistance (Figure 3) [51]. In obesity, macrophage populations in muscle increase and secrete tumour necrosis factor-α (TNF-α), interleukin-1β (IL-1β), and interleukin-6 (IL-6), reinforcing JNK1 and IKKβ activation [51,52]. NLRP3 inflammasome activation within the myofibre links lipids and ROS stress to IL-1β secretion [51,52,53]. TNF-α additionally suppresses PGC-1α expression, reinforcing the mitochondrial biogenesis deficit described in Section 2.4 and further eroding oxidative capacity [54].

*Endoplasmic reticulum and oxidative stress.* Nutrient excess, lipotoxic intermediates, and inflammatory load converge on the endoplasmic reticulum (ER), which is sensitive to saturated fatty acids, ceramides, ROS, and calcium dysregulation [55,56]. Impaired ER calcium handling may be an early feature of insulin-resistant muscle, activating the unfolded protein response (UPR) independently of protein misfolding. Of the three sensors, PERK and IRE1α are most directly implicated: PERK promotes IRS-1 serine phosphorylation, while IRE1α activates JNK1 via tumour necrosis factor receptor-associated factor 2 (TRAF2), making ER stress a further upstream activator of the inflammatory kinase axis rather than a separate pathway (Figure 3) [55,57,58]. ER stress, oxidative stress, and inflammation therefore reinforce one another as a convergence layer feeding back onto the canonical pathway. Complementary evidence from bovine hepatocytes shows that targeting IRE1α and PERK can attenuate fatty-acid-induced insulin resistance [59], supporting conservation of this stress axis across metabolic tissues whilst not constituting direct skeletal-muscle evidence.

*Extracellular matrix remodelling*. The intramuscular ECM is an active signalling environment whose mechanical properties modulate insulin sensitivity, a dimension inaccessible to monolayer culture and one of the strongest arguments for developing 3D EMTs (Figure 3) [60]. In obese and insulin-resistant muscle, increased deposition of collagens I, III, and VI and interstitial fibrosis raise tissue stiffness [61]. Elevated integrin–focal adhesion kinase (FAK) signalling then drives inhibitory IRS-1 serine phosphorylation and disrupts insulin signalling independently of lipid or inflammatory load. Consequently, ECM fibrosis can sustain insulin resistance even when the lipotoxic and inflammatory inputs described in Section 5.1, Section 5.2, Section 5.3 and Section 5.4 are partially resolved [61]. Collagen VI accumulation in the pericellular space correlates with impaired glucose uptake independently of intramyocellular lipid content, and fibrotic stiffening additionally compresses the intramuscular microvasculature, limiting capillary perfusion and insulin delivery (Figure 3). Increased stiffness also suppresses mechanosensitive PGC-1α-dependent programmes, linking ECM remodelling to both mitochondrial and myokine dysfunction. In addition, human skeletal-muscle ECM is highly plastic: exercise training alters locomotor-muscle ECM protein expression [62], whilst fibre-specific ECM alterations have been documented in COPD [63], illustrating matrix remodelling across physiological and disease states and possibly affecting how muscle responds to stimuli.

### 2.5. The Myokine Secretome in Insulin Resistance

Beyond the structural, lipotoxic, mitochondrial, inflammatory, and ECM-mediated mechanisms described above, skeletal muscle insulin resistance is also associated with dysregulation of its endocrine function. Skeletal muscle is a secretory organ that releases myokines in response to contractile and metabolic stimuli, linking muscle activity to local and inter-organ metabolic regulation [10]. Following the recognition of IL-6 as a contraction-induced myokine in 2003, several hundred candidate myokines have been identified through secretome studies, although the biological functions and physiological relevance of many remain incompletely characterised [64,65]. Myokines can act in autocrine, paracrine, and endocrine fashions to influence muscle itself and distant tissues including liver, adipose tissue, pancreas, and brain [10]. Their dysregulation may both reflect the metabolic state of skeletal muscle and contribute to its progression towards insulin resistance [10].

Changes in myokine secretome must be interpreted according to their cellular source, stimulus, and mode of measurement. Muscle gene expression, myofibre secretion, circulating concentration, and contraction-induced release are distinct biological measures and do not necessarily change in parallel. Furthermore, circulating levels can reflect contributions from adipose tissue and immune cells, as well as differences in systemic clearance. This distinction is particularly important for IL-6 and irisin, which illustrate two different challenges in interpreting circulating myokine data.

*Interleukin-6* (IL-6) has divergent effects depending on its source and temporal context: acute contraction-induced muscle IL-6 activates AMPK and promotes GLUT4-dependent glucose uptake, whereas chronic IL-6 derived from adipose and immune cells can impair insulin signalling through SOCS3-mediated inhibition of IRS-1 [10,66]. Total circulating IL-6 therefore cannot distinguish between potentially opposing muscle-derived and inflammatory sources. This limitation is particularly relevant when interpreting IL-6 measurements in both clinical cohorts and experimental co-culture systems, where the cellular origin and duration of IL-6 exposure may differ substantially.

*Irisin* is generated through proteolytic cleavage of fibronectin type III domain-containing protein 5 (FNDC5) and is regulated, at least in part, through PGC-1α-associated pathways. Irisin has been implicated in GLUT4 regulation, mitochondrial biogenesis, and browning of white adipose tissue via uncoupling protein 1 (UCP1) as reviewed in [67]. Exercise has been reported to increase circulating irisin, although the magnitude and consistency of this response vary across studies, and reductions in FNDC5 expression and circulating irisin concentrations have frequently, but not consistently, been reported in T2DM [67,68]. Irisin thus represents a plausible mechanistic link between muscle metabolic state and inter-organ signalling. However, circulating measurements alone do not establish the magnitude or functional importance of muscle-derived irisin, particularly because circulating irisin may reflect differences in both biological regulation and analytical methodology. Methodological differences may account for at least part of the inconsistency across studies. Early human studies commonly relied on commercially available ELISA kits, several of which were subsequently reported to have limited specificity and potential cross-reactivity, raising concerns about the accuracy of the reported circulating irisin concentrations [67]. Jedrychowski et al. [69] subsequently re-examined the detection and quantification of circulating human irisin using quantitative tandem mass spectrometry and reported concentrations substantially lower than those described in many earlier ELISA-based studies. Because ELISA- and mass-spectrometry-based measurements are not directly comparable, differences in reported circulating irisin concentrations between individuals with T2DM and healthy controls should therefore be interpreted in light of the analytical method used [67,68].

The remaining myokines most consistently implicated in insulin resistance follow the same interpretive principles, and their sources, actions, reported changes, and strength of evidence are summarised in Table 1. Briefly:

*Myostatin*, a TGF-β superfamily member and negative regulator of skeletal muscle mass, is associated, in insulin-resistance states, with impaired AKT signalling, reduced GLUT4-mediated glucose uptake, and promotion of ectopic lipid accumulation. It is generally elevated in obesity and T2DM and suppressed by exercise, although the magnitude of this response may vary according to exercise modality, duration, and metabolic context [70,71,72].

*Fibroblast growth factor 21 (FGF21)* is produced by skeletal muscle under conditions of metabolic or mitochondrial stress, but also substantially by the liver. In obesity and T2DM, circulating FGF21 is frequently elevated. This increase has been interpreted as evidence of compensatory signalling and, in some contexts, impaired FGF21 responsiveness [73]. However, elevated circulating FGF21 should not be equated automatically with established FGF21 resistance, since responsiveness is tissue- and context-dependent [73,74].

*Apelin*, a contraction-responsive myokine, activates AMPK and promotes insulin-independent GLUT4 translocation, while supporting mitochondrial metabolism and fatty acid oxidation. Apelin concentrations or expression are frequently reduced with inactivity and obesity, although findings vary across studies and biological compartments [10].

*IL-15* is an exercise-responsive myokine linked to skeletal muscle hypertrophy and adipose tissue regulation. It has also been reported to be reduced with physical inactivity and obesity. Although experimental evidence supports roles for IL-15 in muscle and adipose tissue biology, its direct association with insulin resistance is less well defined than that of IL-6 and myostatin [64].

*Collective remodelling of the secretome.* Taken together, these factors illustrate that insulin resistance is not characterised by a uniform increase or decrease in a fixed set of myokines. The muscle secretome undergoes context-dependent remodelling involving altered contraction-responsive signals, increased stress- or atrophy-associated signalling, and changes in the responsiveness of endocrine pathways. Several myokines, including irisin, apelin, and IL-15, are responsive to muscle activity, but their regulation by exercise, PGC-1α-associated pathways and disease differs [10,64,66]. Meanwhile, myostatin and FGF21 illustrate that signals associated with adverse or compensatory responses may increase or become dysregulated in insulin-resistant states [70,71,72,73,74]. Lipotoxicity, mitochondrial dysfunction, inflammation, ER stress, and ECM remodelling can impair mitochondrial and PGC-1α-associated signalling and reduce physiological contractile activity, providing multiple routes through which the secretory phenotype may become altered.

This context has direct implications for experimental modelling. Because many physiologically relevant myokine responses depend on organised and measurable contractile activity, static systems cannot fully capture the secretory response to muscle function. In addition, several candidate myokines can originate from adipose or immune cells, meaning that circulating measurements alone cannot establish a muscle-specific source. Engineered 3D muscle models therefore provide an opportunity to combine co-cultures with organised contractile activity and a measurement of secretory responses, allowing myokine regulation to be investigated as a functional consequence of muscle physiology rather than solely as a static molecular phenotype. The extent to which current engineered muscle models reproduce these features, and the limitations that remain, is examined in the Sections below.

## 3. Experimental Models of Skeletal Muscle Insulin Resistance

Investigating skeletal muscle insulin resistance requires experimental systems that balance physiological fidelity with mechanistic tractability. The mechanisms outlined in Section 2 define the features an ideal model should capture, yet no single conventional experimental system provides all of them simultaneously. Understanding the strengths and limitations of each model is therefore important for interpreting the literature and identifying how 3D EMTs provide additional experimental value.

### 3.1. Human In Vivo and Ex Vivo Studies

Human skeletal muscle biopsy studies provide direct access to physiologically relevant molecular signatures of insulin resistance. Hyperinsulinaemic-euglycaemic clamp studies combined with vastus lateralis biopsy sampling have provided important mechanistic evidence for impaired insulin-stimulated glucose disposal. More recently, integration with proteomic and phosphoproteomic profiling has deepened insight into the signalling networks involved, identifying individual molecular signatures that predict whole-body insulin sensitivity beyond disease status alone, as described in Section 2.3 [16,17]. Ex vivo approaches including isolated muscle strips and permeabilised fibre bundles extend these studies by enabling controlled pharmacological challenges in human tissue while preserving native fibre architecture [75].

However, the invasive nature of repeated biopsy sampling constrains cohort sizes, limits longitudinal studies, and restricts systematic perturbation experiments. Moreover, biopsy-based studies capture a molecular snapshot of tissue within the systemic context of the donor hormonal milieu, adipose inflammation, hepatic glucose output, and physical activity history. This makes it difficult to distinguish myocyte-intrinsic mechanisms from systemic influences [76]. Human biopsy and clamp studies therefore remain essential for defining the molecular landscape of insulin resistance in vivo but are less suited to controlled causal and mechanistic perturbation.

### 3.2. Rodent and Large Animal Models

Rodent models have been foundational for defining the molecular mechanisms of skeletal muscle insulin resistance under controlled experimental conditions. Diet-induced obesity in C57BL/6 mice and genetic models including ob/ob mice and Zucker diabetic fatty (ZDF) rats recapitulate many features of human metabolic syndrome and have been widely used to investigate insulin signalling, mitochondrial function, pharmacological interventions and inflammation [77,78]. Large animal models, including non-human primates and pigs, provide greater physiological similarity to humans [79].

Nonetheless, important translational limitations apply to skeletal muscle insulin resistance research. Mouse skeletal muscle is composed predominantly of fast glycolytic type IIb fibres, a fibre type that is essentially absent from adult human skeletal muscle, as described in Section 2.1. These fibres differ substantially from the type I and IIa fibres that predominate in human locomotor muscles in terms of metabolic enzyme activity, mitochondrial content and lipid handling [23]. Rodent lipid handling and atherosclerosis susceptibility also differ from those in humans, as discussed in the context of rodent atherosclerosis models [80]. The cardiovascular-model comparisons do not, by themselves, validate skeletal muscle lipotoxicity models. In addition, the rapid development of diabetes in genetic models and the use of supraphysiological dietary fat content do not fully reproduce the gradual, lifestyle-associated trajectory of human insulin resistance [81]. Although many genes and signalling pathways are evolutionarily conserved, specific disease-associated variants and some cytokine systems are not. These species differences need to be considered when extrapolating mechanistic findings to human skeletal muscle.

### 3.3. Two-Dimensional Skeletal Muscle Cultures and Primary Human Myotubes

Conventional 2D skeletal muscle cultures, including immortalised cell lines such as C2C12 and L6, and primary human myotubes derived from biopsy-isolated myoblast precursors are widely used in vitro platforms for mechanistic studies of insulin resistance. They offer experimental accessibility, cost-effectiveness, and compatibility with high-throughput pharmacological and genetic manipulation under controlled metabolic conditions. Primary human myotubes can retain important donor-specific metabolic characteristics, including aspects of the donor’s insulin sensitivity and lipid handling phenotype, making them valuable for investigating cell-autonomous mechanisms of insulin resistance.

Many processes previously considered accessible only to more complex models can be studied in 2D culture. Electrical pulse stimulation (EPS) can induce contractile activity and contraction-regulated myokine release, while fatty-acid loading, cytokine exposure, mitochondrial respirometry, and insulin-stimulated glucose uptake are well established in these systems. The principal limitation of 2D culture is therefore not the inability to study these individual processes, but their limited integration within a physiologically organised tissue. Myotubes cultured on rigid plastic substrates do not develop mature sarcomeric architecture, fibre-type identity, or aligned myofibrillar structure. Consequently, integrin-mediated mechanosignalling, matrix stiffness, force production, and coordinated integration of contractile, metabolic, and secretory responses are only partially represented [2,12]. Cellular diversity is also typically limited. Thus, 2D cultures remain highly useful for defined mechanistic questions, but provide a less integrated representation of skeletal muscle physiology than 3D systems.

Two-dimensional systems therefore remain the most appropriate choice for many applications, including high-throughput screening, genetic manipulation, and the dissection of individual signalling steps. Engineered 3D models are not required for all experimental questions. Rather, the two approaches are complementary: 2D culture offers accessibility and scalability at lower cost, whereas 3D systems provide greater tissue-level integration when that integration is the object of study.

### 3.4. iPSC-Derived Myogenic Models

Human induced pluripotent stem cell (iPSC)-derived myogenic models provide a renewable, patient-specific cell source without repeated biopsy sampling. Myotubes differentiated from donors with T2DM retain disease-associated defects in insulin-stimulated glucose uptake and mitochondrial oxidative function following reprogramming, suggesting that at least a component of the insulin-resistant phenotype may be retained through epigenetic mechanisms [82,83]. Isogenic comparisons between corrected and uncorrected disease lines also provide a level of genetic control that is difficult to achieve with primary cultures.

However, current differentiation protocols generally produce metabolically immature cells with variable fibre-type specification that do not reliably reproduce the type I/IIa composition of adult locomotor muscle. Moreover, when maintained in conventional 2D formats, they share the architectural limitation of primary cultures, including limited development of mature contractile organisation and tissue-level integration of contraction-regulated responses [83]. Their potential for modelling insulin resistance may therefore be greatest when patient-specific cells are incorporated into 3D systems.

### 3.5. Comparative Summary

Conventional systems provide complementary levels of physiological relevance and experimental control, but none simultaneously integrates a human genetic background, organised three-dimensional architecture, contractile function, ECM-mediated mechanosignalling, and contraction-regulated myokine secretion within a single tissue. The distinguishing feature of 3D EMT is therefore not superiority across individual experimental parameters, but the ability to integrate multiple aspects of skeletal muscle physiology within one construct. This provides the rationale for its application to insulin resistance.

Table 2 compares experimentally relevant features across commonly used models of skeletal muscle insulin resistance. Because 3D EMT encompasses systems with different cell sources and levels of complexity, these models are stratified accordingly. Ratings describe the typical capabilities of each model class rather than fixed properties of every individual implementation. The strengths and limitations of each model are summarised in Table 2. Although functional neuromuscular junctions and vascularisation/perfusion have been achieved in specialised engineered neuromuscular and sensorimotor systems, they have not yet been incorporated into metabolic 3D EMTs and are summarised as such in Table 2. The information in this Table reflects the broader capabilities of 3D EMT rather than established features of current insulin-resistance models.

## 4. Three-Dimensional Engineered Skeletal Muscle: Capabilities and Demonstrated Applications

Three-dimensional EMTs have emerged from the convergence of biomaterials science, stem cell biology, and tissue engineering, driven in part by the limitations of conventional models described in Section 3. Their value lies not in superiority over 2D systems per se, but in their ability to combine organised myofibrillar architecture, measurable contractile function, ECM-mediated mechanosignalling, and accessible secretome within a single construct. This enables several mechanisms relevant to insulin resistance, which are often studied separately in conventional models, to be investigated within a more integrated tissue context. This section describes these capabilities while distinguishing experimentally demonstrated applications from those that remain promising but have not yet been extensively established.

### 4.1. Design Principles and Scaffold Architecture

EMTs are typically constructed by embedding myogenic cells within a hydrogel scaffold cast around mechanical anchor points, typically two fixed posts within a mould, which provide uniaxial tension along the longitudinal axis of the construct [84]. This tensional environment promotes myoblast alignment, fusion into elongated myotubes, and the development of organised myofibrillar architecture that is largely absent in monolayer culture. Two fabrication strategies are commonly used. In self-organisation approaches, a suspension of cells and hydrogel is cast into a mould and progressively compact around the anchors as tension develops [85]. In bioprinting approaches, cells are deposited in defined spatial patterns within or between hydrogel layers, enabling greater control over fibre orientation, construct geometry, and multi-cellular organisation [86].

Scaffolds are most commonly formed from fibrin or collagen type I. Collagen type I is a major structural component of native skeletal muscle ECM and supports myoblast proliferation and differentiation, whereas fibrin occurs naturally as a scaffold in native muscle repair and can promote myogenic markers but is susceptible to rapid proteolytic degradation during extended culture, potentially compromising construct integrity in long-term metabolic studies [87]. Matrigel is also frequently incorporated to enhance cell adhesion and differentiation, although notorious batch-to-batch variability limits reproducibility [88]. These scaffolds should be considered mimics of selected biophysical and biochemical features of the native ECM rather than faithful reproductions of the native matrix environment. Nevertheless, because scaffold stiffness can modulate integrin-FAK signalling and thereby influence insulin sensitivity (Section 2.4), scaffold composition represents an experimentally tuneable variable. This feature is difficult to manipulate systematically in monolayer culture and contributes to the potential value of 3D systems for investigating the mechanobiological contribution to insulin resistance.

### 4.2. Cell Sources and Their Relevance to Insulin Resistance Modelling

The choice of cell source determines both the maturation state of the construct and its translational relevance to human insulin resistance and should be matched to the specific research question.

*C2C12 murine myoblasts* remain the most widely used cell source due to their robust, reproducible myogenic capacity [85]. In 3D constructs, they reliably form aligned, contractile myotubes suitable for proof-of-concept studies and pharmacological screening, as demonstrated by Na et al. [15]. However, they are predisposed towards fast-twitch phenotypes and retain the species-specific limitations discussed in Section 2.1 and Section 3.2. Their use is therefore particularly appropriate for initial platform validation and mechanistic studies rather than investigations requiring human translational relevance.

*Primary human myoblasts* provide a physiologically relevant model of human skeletal muscle and can exhibit increased maturation in 3D compared to matched 2D cultures [84]. They can also retain donor-specific metabolic traits, including insulin sensitivity, lipid handling, and oxidative capacity, enabling the investigation of cell-autonomous mechanisms and inter-individual metabolic variability [89]. However, their limited proliferative capacity and variability between donors can hinder experimental scalability and reduce throughput. Further background on primary human skeletal muscle culture and muscle development is provided in [90,91].

*Human iPSC-derived myogenic progenitors* provide a potentially renewable, patient-specific cell source with particular advantage for isogenic experimental designs and personalised phenotyping linked to individual molecular signatures (Section 2.3). However, current differentiation protocols generally produce immature, fast-twitch-biassed myotubes that do not consistently reproduce the oxidative phenotype of insulin-sensitive adult human skeletal muscle [92]. This limitation is particularly relevant to myokine studies, because PGC-1α-dependent FNDC5/irisin expression is associated with oxidative muscle maturation (Section 2.5).

Regardless of cell source, fibre-type composition can be partially influenced by culture conditions. For example, chronic electrical pulse stimulation (EPS) can promote a shift towards slower more oxidative phenotype, thereby recapitulating aspects of the exercise-trained state (Section 4.3) [93].

### 4.3. Contractile Function and Calcium Handling

A key functional feature of 3D EMTs is their ability to generate measurable force, providing a direct link between metabolic processes and contractile output. Under EPS, 3D constructs generate quantifiable twitch and tetanic forces, enabling systematic comparison across experimental conditions [94,95]. This is relevant because, as described in Section 2.2, contractile activity independently regulates GLUT4 trafficking through the AMPK–TBC1D1 axis and stimulates contraction-regulated myokine secretion. Force generation can therefore provide an integrated functional readout alongside metabolic and endocrine measurements. Although contractile activity and myokine secretion can also be induced in 2D cultures using EPS (Section 3.3), the value of 3D systems lies in the ability to assess force generation, glucose handling, and secretome within the same organised tissue construct rather than as independent features of separate assays.

Calcium transients provide a complementary measure of excitation–contraction coupling and can be visualised in EMTs using indicators such as Fluo-4 AM [96]. This readout is relevant because contraction-linked calcium signalling contributes to both force generation and the CaMKII–dependent component of GLUT4 trafficking described in Section 2.2 [34,35]. Because impaired calcium handling is increasingly recognised as a feature of insulin-resistant muscle, 3D constructs provide an opportunity to examine its functional consequences, including changes in force generation and contraction-associated glucose uptake, within the same experimental system.

Chronic EPS of human 3D EMTs in the Biowire II platform has been shown to reproduce several exercise-associated adaptations, including fast-to-slow fibre-type transition, myotube hypertrophy, increased fatigue resistance, and changes in force profiles resembling those observed in trained muscle [93]. This provides a functional context in which exercise-related processes described earlier, including AMPK-mediated sensitisation, contraction-dependent irisin and apelin secretion (Section 2.5), can be investigated within an integrated contractile tissue rather than inferred solely from pharmacological activation of AMPK.

### 4.4. Functional Assays and Demonstrated Models of Insulin Resistance

Functional assessment of insulin resistance in 3D EMTs should extend beyond morphological characterisation to demonstrate altered insulin-responsive metabolic function [15]. A principal readout is insulin-stimulated glucose uptake, assessed using approaches such as 2-NBDG or radiolabelled 2-deoxyglucose under basal, insulin-stimulated, and insulin-resistant conditions. Ideally, this should be paired with insulin signalling endpoints (IRS-1, AKT, AS160/TBC1D4 phosphorylation, and GLUT4 localisation) together with viability measures to distinguish true insulin responsiveness from poor differentiation or tissue viability [97]. A further strength of 3D systems is the potential to combine these measurements with contractile force, mitochondrial respiration, lipidomics profiling of DAG and ceramide species, and conditioned-medium analysis of myokine secretion within the same construct.

The number of studies that have applied genuine 3D constructs to insulin resistance remains small and their scope should therefore be described precisely. Na et al. [15] developed a C2C12-based construct in an alginate-collagen scaffold in which 0.1 mM palmitate reduced insulin-stimulated 2-NBDG glucose uptake while maintaining cell viability. The model exhibited a stronger insulin response than matched 2D cultures. It was rescued by rosiglitazone and metformin and identified screening hits with greater concordance with ex vivo mouse soleus responses than hits selected using 2D cultures [15]. This provides a well-characterised demonstration of lipid-induced insulin resistance and drug screening, although its reliance on murine C2C12 cells limits direct human translational relevance. Other 3D studies have reported metabolic abnormalities associated with diabetic donors [98], altered insulin-stimulated glucose uptake associated with adipogenesis [99], and fatty acid-induced lipid accumulation accompanied by impaired muscle function [100].

Two studies have modelled adipocyte–muscle interactions relevant to myosteatosis. One study used a spatially controlled construct to show that direct versus paracrine adipocyte–muscle interactions differentially affected lipid accumulation and metabolic activity. The second study developed a vascularised adipose-muscle construct in which increasing adipogenic content was associated with elevated basal but blunted insulin-stimulated glucose uptake [99,101]. A separate line of work has investigated GLUT4-overexpressing constructs as a therapeutic strategy, demonstrating improved glycaemic control on implantation in diabetic mice and EMTs incorporating GLUT4 overexpression [102,103]. These studies demonstrate that 3D EMTs can reproduce aspects of lipid-induced and myosteatosis-associated insulin resistance and can engage in functional interactions with other metabolically relevant tissues. However, the evidence base remains small and is predominantly non-human. A broader review on human tissue-engineered skeletal muscle as a metabolic research platform is provided in [104].

### 4.5. Validation Against Human Tissue

An important and often under-addressed question is the extent to which 3D EMTs reproduce human skeletal muscle at the molecular and functional levels. Current validation evidence is encouraging but remains limited. At the functional level, EPS-conditioned human constructs reproduce exercise-associated changes in fibre type and force generation that parallel adaptations observed in trained human muscle [93]. At the molecular level, the most direct comparison to date comes from the inflamed adipose–macrophage–myobundle microphysiological system described in Section 5.3, in which co-culture induced transcriptomic changes reported to resemble those observed in obese human muscle [105], providing a point of correspondence, albeit a qualitative one. Primary human and iPSC-derived muscle models can additionally retain donor- or disease-associated metabolic characteristics, offering a further point of correspondence with individual in vivo phenotypes [83,89].

However, systematic and quantitative benchmarking remains limited. Current validation relies on a relatively small number of targeted comparisons rather than formal equivalence between engineered and native human muscle. Adequate validation would require several elements that have not yet been established together: quantitative comparison of transcriptomic and proteomic profiles against matched human skeletal muscle, with predefined concordance metrics and appropriate control of batch and technical effects, rather than qualitative resemblance alone; direct comparison of insulin-signalling and metabolic outputs, including insulin-stimulated glucose uptake and downstream signalling; calibration of functional measures such as force generation, contractile responses, mitochondrial function, and myokine secretion against human values; the use of sufficiently large and diverse donor cohorts to capture reproducibility and inter-individual variation; and, where feasible, validation across independent platforms and laboratories. To our knowledge, no study has yet demonstrated all these elements within a single, comprehensive validation framework. Establishing such benchmarks and distinguishing clearly between evidence of correspondence and formal validation should therefore be an important priority for the field.

## 5. What Cannot Yet Be Modelled: Limitations and Open Challenges

The capabilities described in Section 4 should be read alongside an equally explicit account of what current 3D EMT cannot yet achieve. Several of the most important aspects of skeletal muscle insulin resistance remain beyond present platforms, either because key structural features are missing or because the enabling experiments have not been performed. Distinguishing these unrealised applications from demonstrated ones is essential to a fair appraisal of the field.

### 5.1. Structural and Physiological Limitations

*Fibre maturation.* Even the most advanced constructs remain metabolically and structurally immature relative to adult human skeletal muscle. Fibre-type specification remains incompletely controlled, and many constructs are biassed towards immature and fast-twitch phenotypes, with under-representation of the oxidative type I and IIa fibres that are particularly relevant to insulin sensitivity (Section 4.2). Because oxidative maturation contributes to PGC-1α-dependent processes, including mitochondrial biogenesis and FNDC5/irisin expression, this immaturity limits the extent to which current models can capture the mitochondrial and myokine axes described in Section 2.4 and Section 2.5.

*Neuromuscular junctions.* In vivo, motor innervation contributes to fibre-type specification, myosin heavy chain expression, and metabolic phenotype [106]. In most current metabolic EMTs, contraction is instead induced by exogenous EPS rather than autonomous neural activation. Consequently, these models do not reproduce the full contribution of motor neuron activity to muscle metabolism, limiting their ability to investigate metabolic consequences of altered neuromuscular activity relevant to ageing, denervation, and diabetic peripheral neuropathy. Motor neuron-derived influences on PGC-1α expression and myokine regulation are likewise not captured [107,108]. Functional NMJs have been demonstrated as proof-of-concept in engineered neuromuscular and sensorimotor systems, including some human constructs used for neuromuscular disease modelling [109,110,111]. However, this capability has not been integrated into metabolic 3D muscle models or applied to insulin resistance, and findings from NMJ-absent constructs should be interpreted within this limitation.

*Vascularisation and perfusion.* Most current constructs lack a mature vascular network and rely predominantly on diffusion for oxygen and nutrient delivery. This limits construct size and can result in hypoxic or nutrient-deprived regions in larger tissues. These limitations are particularly relevant to metabolic studies because hypoxia or inadequate nutrient delivery can alter glucose and lipid metabolism independently of insulin resistance, potentially confounding metabolic readouts. Microfluidic and vascularised designs are beginning to address these challenges, and vascularised muscle constructs have been developed [99]. In a separate study, bioprinted 3D EMT was shown to undergo vascularisation and innervation following implantation [96]. However, these findings do not establish controlled physiological perfusion in an insulin resistance model. Achieving robust, physiologically relevant perfusion remains challenging and is not yet a routine feature of metabolic 3D EMT models. Further development of vascularised and perfused platforms will therefore be important for enabling larger constructs and improving the reliability of quantitative metabolic measurements.

### 5.2. Mechanisms Not Yet Investigated in 3D EMTs

Beyond these structural limitations, several central features of insulin resistance remain insufficiently investigated in 3D human EMTs. No published study has used a 3D EMT derived from primary human or iPSC-derived muscle cells to interrogate the canonical and non-canonical insulin signalling defects identified by phosphoproteomics, including MINDY1, mTORC1 impairment, and the broader network disruptions described in Section 2.2 and Section 2.3. Similarly, the mechanobiological contribution to insulin resistance described in Section 2.4 has not been systematically tested by manipulating scaffold stiffness in a human 3D insulin-resistance model, despite the potential of 3D systems to control this parameter experimentally. Exercise-mimicking EPS has not, to our knowledge, been combined with an insulin resistance induction protocol in a human 3D construct to examine the AMPK–GLUT4 axis alongside contraction-regulated myokine secretion within the same experimental system. Finally, although multi-tissue microphysiological systems incorporating muscle, adipose and inflammatory components have been developed, integration with liver and adipose modules to model the inter-organ lipid, inflammatory, and myokine interactions characteristic of systemic insulin resistance remains limited. These gaps define important opportunities for future investigation rather than established applications of current 3D EMTs.

### 5.3. The Myokine Axis: A Demonstrated Capability with Unrealised Potential

The myokine dimension illustrates both the promise and the current limitations of 3D muscle. Because physiological myokine secretion depends on organised contractile activity and, for some factors, on cellular source (Section 2.5), it is particularly amenable to systems that combine controlled contraction with defined cellular composition. Direct evidence already exists. Sugimoto et al. showed that EPS of C2C12-based 3D constructs increased irisin secretion without altering cathepsin B, providing evidence that contraction can differentially regulate these secreted factors [12]. In a separate microphysiological system, Kim et al. [105] combined differentiated human myotubes and macrophages within a human 3D muscle construct. This co-culture demonstrated reduced contractility, increased levels of a defined cytokine panel, and transcriptomic changes that were reported to resemble those observed in obese human muscle [105].

These studies provide proof-of-principle, but they also highlight several unanswered questions. For example, it remains unclear whether the blunted irisin response reported in insulin-resistant individuals reflects a cell-autonomous defect in contraction–secretion coupling, an extrinsic inflammatory influence, or both. Similarly, whether myostatin-mediated suppression of AKT signalling can be pharmacologically modulated in a system in which myokine secretion and downstream signalling are measured together remains to be established. The extent to which apelin can influence AMPK-mediated GLUT4 translocation independently of the IRS-1/AKT axis (Section 2.2) also remains unresolved in an integrated 3D human model. More broadly, whether patient-derived iPSC constructs can reproduce individual differences in myokine secretion and link these phenotypes to non-canonical signalling nodes such as MINDY1 has not yet been demonstrated. These questions are experimentally approachable, but the necessary integrated models have not yet been established.

The ability to combine myokine profiling with glucose uptake, force generation, respirometry, and lipidomics measurements within a single construct therefore represents an emerging opportunity rather than an established application. This distinction is important when considering the translational potential of 3D EMT models: the technical capacity to measure several relevant outputs does not, by itself, demonstrate that these measurements can yet be integrated into a validated model of human insulin resistance.

### 5.4. Clinical Translation Gap

A long-term goal of patient-specific 3D modelling is to predict individual disease phenotypes and treatment responses. However, current evidence remains insufficient to support this level of clinical translation. To date, findings have been more limited: primary human muscle cells can retain donor-specific metabolic characteristics [89], iPSC-derived constructs offer opportunities for patient-specific disease modelling [83], and a microphysiological system has reproduced gene-expression features associated with obese human muscle [105]. Although these findings represent important steps towards personalised modelling, they do not demonstrate individual-level phenotype prediction. Specifically, it remains to be established whether constructs derived from an individual patient can recapitulate their insulin-resistance phenotype, predict treatment response, or identify the predominant pathogenic mechanisms underlying their disease. Until such validation is available, the precision-medicine potential of these platforms should be presented as a scientifically motivated objective rather than as an established clinical capability.

## 6. What Can and Cannot Yet Be Modelled: A Synthesis

The preceding sections establish the mechanisms through which skeletal muscle becomes insulin resistant (Section 2) and the capabilities and limitations of current experimental systems (Section 3, Section 4 and Section 5). Bringing these together allows the central question of this review to be addressed directly: which aspects of skeletal muscle insulin resistance can currently be investigated in 3D EMTs, which can be investigated only partially, and which have not been systematically modelled. Table 3 provides this mapping, distinguishing throughout between experimentally demonstrated applications and those that remain plausible but insufficiently established.

Three broad conclusions emerge. First, the aspects most readily captured are those that are relatively cell-autonomous or can be induced by defined metabolic perturbations, including insulin-stimulated glucose uptake, lipid-induced insulin resistance, and contractile function. However, much of the available evidence derives from murine C2C12-based constructs rather than human engineered muscle, limiting direct translational interpretation. Second, several features for which 3D systems offer important experimental opportunities, including ECM mechanobiology, adipose–muscle crosstalk, and contraction-regulated myokine secretion, remain only partially established, often based on individual proof-of-concept studies. Third, several clinically and mechanistically important questions have not yet been systematically addressed in human 3D muscle, including patient-specific phosphoproteomic signatures, the combined effects of exercise-like contraction and insulin resistance, and the integration of muscle with vascular, neural, and multi-organ components. These should therefore be regarded as priorities for further development rather than established capabilities of current platforms.

## 7. Conclusions

Skeletal muscle insulin resistance arises not from a single lesion but from an integrated network spanning canonical and non-canonical signalling, lipotoxicity, mitochondrial and ER stress, inflammation, ECM remodelling, and dysregulation of the myokine secretome. Capturing this integration, rather than any individual pathway in isolation, is the central challenge for experimental models. As summarised in Section 6 and Table 3, 3D engineered muscle has demonstrated genuine but partial capabilities in meeting this challenge; its principal value lies not in surpassing conventional models, but in integrating structural, contractile, metabolic, and endocrine readouts within a single human tissue context, a potential that remains, for the most part, to be realised.

Progress will depend on addressing these limitations. An immediate priority is improved fibre maturation together with systematic validation of constructs against matched human muscle, since without robust molecular and functional benchmarks the fidelity of individual models remains difficult to establish. Building on this foundation, incorporation of perfusable vascularisation could reduce the diffusion constraints that currently limit construct size and may confound metabolic readouts, while functional innervation would enable more physiologically relevant regulation of contraction and myokine secretion. In parallel, patient-derived iPSC constructs linked to individual phosphoproteomic and myokine profiles could provide a route towards personalised metabolic phenotyping. Their eventual integration with adipose, liver, and immune modules in multi-tissue microphysiological systems would allow the inter-organ crosstalk characteristic of systemic insulin resistance to be studied in a controlled human context. Realising this trajectory would move 3D EMTs from a promising in vitro model towards a more comprehensive platform for mechanistic investigation, therapeutic screening, and, ultimately, precision metabolic medicine.

## Figures and Tables

**Table 1:** Myokines relevant to skeletal muscle insulin resistance: sources, metabolic actions, reported changes, and strength of evidence.

Myokine	Principal Source & Regulation	Main Metabolic Actions	Reported Change in Obesity/T2DM	Strength of Evidence
IL-6 [10,55,65]	Contracting muscle (acute) Adipose tissue Macrophages (chronic)	Acute muscle-derived IL-6 can activate AMPK and promote GLUT4-dependent glucose uptake, anti-inflammatory. Chronic adipose/immune-derived IL-6 can promote SOCS3-mediated suppression of IRS-1 signalling	Chronically elevated in obesity/T2DM, particularly with adipose/immune contribution; acute muscle-derived response to exercise may be blunted	Strong: source- and duration-dependent effects are supported by consistent experimental and physiological evidence, although the net metabolic effects depend on cellular source and exposure context
Irisin (FNDC5) [67,69,68]	Skeletal muscle; associated with PGC-1α signalling and exercise/contraction	Reported to promote GLUT4 expression, mitochondrial biogenesis, and white adipose browning via UCP1	Frequently reported as reduced in obesity/T2DM, but findings are inconsistent across human studies	Moderate mechanistic evidence; limited and method-dependent human biomarker evidence: FNDC5/irisin biology is experimentally supported but circulating irisin measurements differ substantially between ELISA- and mass-spectrometry-based assays, so reported human differences should be interpreted cautiously.
Myostatin [70,71,72]	Skeletal muscle; TGF-β superfamily	Negative regulator of muscle mass; suppresses AKT associated anabolic signalling and can impair GLUT4 related metabolic pathways; promotes adipogenic processes	Frequently reported as elevated and inversely associated with insulin sensitivity and muscle mass	Strong mechanistic evidence; moderate human association evidence: the role of myostatin as a negative regulator of muscle growth is well established, whereas the magnitude and direction of its association with insulin resistance vary with population and metabolic context
FGF21 [73,74]	Primarily liver; also produced by skeletal muscle under metabolic nutrient and mitochondrial stress	Promotes fatty acid oxidation, thermogenesis, adiponectin signalling and can limit accumulation of lipotoxic intermediates	Often elevated in obesity/T2DM and associated with metabolic dysfunction; elevation has been interpreted as compensatory response and/or as evidence of reduced FGF21 responsiveness	Moderate: repeated elevation has been reported in metabolic disease, but its interpretation is context-dependent and may reflect both increased production and impaired downstream responsiveness
Apelin [10]	Skeletal muscle; APLN gene expression and contraction-associated regulation	Activates AMPK, can promote GLUT4 translocation independently of insulin, and may support mitochondrial biogenesis	Frequently reported as reduced in obesity/T2DM, but findings vary between studies and tissues	Moderate: mechanistic evidence supports metabolic actions, but human evidence for altered apelin concentrations or expression in obesity/T2DM is less consistent
IL15 [65]	Skeletal muscle; exercise-associated regulation	Promotes muscle hypertrophy and may reduce adipose mass via endocrine crosstalk	Reported to be reduced with physical inactivity and obesity	Limited-to-moderate: experimental evidence supports roles in muscle and adipose biology, but evidence linking altered IL-15 directly to human insulin resistance is less consistent

Strength-of-evidence: Evidence categories consider (i) consistency across independent studies, (ii) directness of evidence linking the myokine to the reported metabolic effect, (iii) reproducibility and specificity of measurement methods, and (iv) biological context, including species, cellular or tissue source, exercise status, disease state, and exposure duration. Strong indicates relatively consistent mechanistic evidence supported by complementary physiological or human evidence; moderate indicates reproducible evidence in at least one domain but with important limitations in consistency, directness, or translational validation; and limited-to-moderate indicates plausible mechanistic support with comparatively limited or heterogeneous human evidence. These categories are qualitative and describe the current evidence base rather than representing a formal quantitative evidence grade.

**Table 2:** Comparative capabilities of experimental models for skeletal muscle insulin resistance.

Feature	Human Biopsy/Ex Vivo	Rodent/ Large Animal	2D Myotubes	2D iPSC-Derived	3D EMT Rodent	3D EMT Human Primary/iPSC	3D MPS
Human genetic background	✓	–	✓	✓	–	✓	✓
Organised 3D myofibre architecture	✓	✓	–	–	✓	✓	✓
Measurable contractile function	✓	✓	±	±	✓	✓	✓
ECM organisation	✓	✓	–	–	±	±	±
Mechanosignalling	✓	✓	±	±	±	✓	✓
Neuromuscular integration	±	✓	–	–	–	±	±
Vascular/perfusion context	±	✓	–	–	–	–	±
Contraction regulated myokine release	✓	✓	±	±	✓	✓	✓
Adipose/immune crosstalk	✓	✓	–	–	–	±	✓
Controlled mechanistic perturbation	±	✓	✓	✓	✓	✓	✓
Throughput/scalability	–	±	✓	✓	✓	±	±

Rating criteria: ✓ generally represented or experimentally achievable; ± possible in specialised implementations or dependent on the experimental configuration; – generally not represented or readily achievable. For neuromuscular integration and vascular/perfusion context, “±” indicates that the feature has been demonstrated in specialised engineered neuromuscular or vascularised systems but has not yet been incorporated into metabolic models. MPS: microphysiological systems are 3D muscle constructs, which incorporate additional features such as multiple tissue types, microfluidic perfusion, or vascularisation to reproduce tissue- and organ-level function with greater fidelity. Ratings indicate typical capabilities of the model class and do not imply equivalent physiological fidelity across models.

**Table 3:** Mechanisms of skeletal muscle insulin resistance and current 3D EMT modelling status.

Mechanism/Feature (Section)	Modelling Status	Evidence/Representative Study	Principal Barrier or Caveat
Insulin-stimulated glucose uptake (Section 2.2)	Demonstrated	Palmitate-induced insulin resistance with 2-NBDG uptake with pharmacological rescue reported in a 3D construct [15].	Evidence is predominantly from murine C2C12 constructs; validation in human engineered muscle remains limited.
Lipotoxicity/lipid-induced insulin resistance (Section 2.4)	Demonstrated	Fatty-acid exposure has been used to induce impaired insulin-stimulated glucose uptake and lipid accumulation in 3D muscle constructs [15,100].	Primarily reflects a defined, cell-autonomous metabolic challenge and does not reproduce systemic lipid overflow or multi-organ metabolic interactions.
Myosteatosis/adipocyte–muscle crosstalk (Section 4.4)	Demonstrated (partially)	Diabetic-donor-derived constructs exhibit metabolic and structural abnormalities [98]; vascularised adipogenic muscle constructs demonstrate altered insulin-stimulated glucose uptake [99]; a 3D co-culture platform enables investigation of direct and indirect adipocyte–muscle interactions [101].	Evidence remains limited and construct-dependent; patient-derived and systematically validated models are lacking.
Contractile/calcium function (Section 4.3)	Demonstrated	Force generation and Ca^2+^ transients quantifiable; while chronic EPS has been used to induce changes in fibre phenotype and contractile properties [93,94,110].	Maturation generally remains below that of adult human skeletal muscle, particularly with respect to fibre-type composition and metabolic phenotype.
Inflammatory adipose–muscle signalling (Section 2.4)	Demonstrated (single system)	Inflamed adipose–macrophage–myobundle MPS reproduces cytokine profile and insulin-resistance gene signature [105].	One platform to date; human validation preliminary.
ECM mechanobiology/stiffness (Section 2.4)	Partially	Fibrin hydrogel-based functional muscle constructs have been developed [87]; their use establishing a causal link between matrix stiffness and induced insulin remains unvalidated.	The causal contribution of matrix stiffness to insulin resistance has not yet been systematically tested in a human 3D insulin-resistance model.
Contraction-regulated myokine secretion (Section 2.5)	Partially	EPS has been shown to increase irisin secretion in a 3D C2C12 construct [12]; secretome accessible in conditioned medium.	Integration with insulin-resistance induction, human patient-derived constructs, and physiologically regulated contraction remains limited.
Non-canonical/phosphoproteomic signatures, including MINDY1 (Section 2.2 and Section 2.3)	Not yet systematically investigated	No 3D human construct has interrogated these signalling pathways.	Requires patient iPSC constructs and phosphoproteomic workflows.
Combined exercise like contraction (EPS) and insulin-resistance induction (Section 4.3)	Not yet demonstrated in human 3D muscle	Exercise-like EPS and insulin-resistance induction can each be modelled separately, but their combination has not, to our knowledge, been systematically investigated in a human 3D construct [12,15].	Requires integration of contraction, insulin-resistance induction, metabolic phenotyping, and myokine profiling within the same experimental system.
Neuromuscular junction/innervation (Section 5.1)	Emerging, but not yet integrated into metabolic models	Functional NMJs have been demonstrated in specialised neuromuscular organoids and engineered muscle systems; however, their application to metabolic disease modelling remains largely unexplored [109,110].	Early-stage integration into insulin-resistance models; effects of autonomous neural activity on metabolism, fibre phenotype, and myokine secretion remain insufficiently characterised.
Vascularisation/perfusion (Section 5.1)	Emerging, but not yet routine in metabolic models	Vascularised and adipogenic muscle constructs have been developed [99]; bioprinted muscle constructs have shown vascularisation following implantation [96]; controlled physiological perfusion has not been demonstrated in a human 3D insulin-resistance model.	Robust physiological perfusion remains challenging, and most metabolic constructs still rely predominantly on diffusion, creating potential size, oxygenation, and nutrient-delivery constraints.
Multi-tissue (muscle–liver–adipose) integration (Section 5.2)	Not yet established for systemic insulin resistance	Adipocyte–muscle and inflamed adipose–muscle co-culture systems have been reported [101,105]; a fully integrated muscle–liver–adipose model of systemic insulin resistance has not been demonstrated.	Defines the next frontier
Patient-specific phenotype prediction (Section 5.4)	Not yet demonstrated; (aspirational)	Donor metabolic memory retained, but phenotype prediction not demonstrated [89].	Requires quantitative validation against matched patient-level molecular and functional data before precision-medicine claims can be supported

Modelling status: “demonstrated” indicates the feature has been shown experimentally in at least one 3D engineered muscle study; “partially” indicates preliminary or single-study evidence, or demonstration in a related context; “not yet” indicates a plausible but so far unrealised application. Several demonstrated capabilities derive from murine (C2C12) rather than human constructs, as noted.

## Data Availability

No new data were created or analyzed in this study. Data sharing is not applicable to this article.

## Abbreviations

AKT, protein kinase B; AMPK, AMP-activated protein kinase; AS160/TBC1D4, AKT substrate of 160 kDa/TBC1 domain family member 4; CaMKII, calcium/calmodulin-dependent protein kinase II; DAG, diacylglycerol; ECM, extracellular matrix; ECAR, extracellular acidification rate; 3D, three- dimensional; EMT, 3D engineered muscle tissue; EPS, electrical pulse stimulation; ER, endoplasmic reticulum; FAK, focal adhesion kinase; FGF21, fibroblast growth factor 21; FNDC5, fibronectin type III domain-containing protein 5; GLUT4, glucose transporter type 4; IKKβ, inhibitor of κB kinase β; IL, interleukin; iPSC, induced pluripotent stem cell; IR, insulin receptor; IRS-1, insulin receptor substrate-1; JNK, c-Jun N-terminal kinase; MASLD, metabolic dysfunction-associated steatotic liver disease; MPS, microphysiological systems; mTORC1/2, mammalian target of rapamycin complexes 1/2; NMJ, neuromuscular junction; OCR, oxygen consumption rate; PGC-1α, peroxisome proliferator-activated receptor gamma coactivator 1-alpha; PI3K, phosphoinositide 3-kinase; ROS, reactive oxygen species; T2DM, type 2 diabetes mellitus; TNF-α, tumour necrosis factor-α; UPR, unfolded protein response.
